# Psychiatric symptoms and their predictors in aging parents of adults with autism spectrum disorder

**DOI:** 10.1038/s41598-025-00124-0

**Published:** 2025-09-26

**Authors:** Müjdat Erarkadaş, Kübra Özmeral Erarkadaş, Şahika Gülen Şişmanlar

**Affiliations:** 1 Clinic of Child and Adolescent Psychiatry, Gölcük Necati Çelik State Hospital, Kocaeli, Turkey; 2 Child and Adolescent Psychiatry Department, Medical Faculty, Kocaeli University, Kocaeli, Turkey

**Keywords:** Autism spectrum disorder, Adult, Parents, Psychiatric symptom, Psychopathology, Autism spectrum disorders, Human behaviour

## Abstract

Parenting an individual with Autism Spectrum Disorder (ASD) affects the mental health of both mothers and fathers. A chronic disorder, ASD, has devastating effects on parental mental health as the affected individual moves from childhood to adulthood. While the effects of ASD on parental mental health during childhood have been studied extensively, there is limited information regarding the mental health of aging parents of the expanding adult ASD population. In this context, we aimed to determine the psychiatric symptoms (PS) levels of parents of adult with ASD, to compare the PS level between mothers and fathers, to investigate the relationship between parental PS and variables related to the individuals with ASD and their parents. To assess the parents’ PS, the Brief Symptom Inventory was administered to 77 parents of adults with ASD. ASD severity was evaluated with the Childhood Autism Rating Scale, behavioral problems were assessed with the Aberrant Behavior Checklist, independence level (IL) of the cases was measured with the Lawton Instrumental Activities of Daily Living Scale, and social functioning level (SFL) of the cases was evaluated using the Social Functioning Scale. At all ages from childhood to adulthood, the most common primary caregiver was mother. Mothers’ labor force participation rate was significantly lower than fathers’ (*p <* 0.05). Mothers’ somatization (*p* = 0.028) and depression (*p* = 0.002) levels were significantly higher than fathers’. The somatization score of the mothers of cases with comorbid medical diagnosis and intellectual disability (ID) was significantly higher than those without. The depression score of fathers of cases with ID and illiteracy was significantly higher (*p <* 0.05). The negative self-concept score of fathers of cases with ID, illiteracy, and dependent self-care and toileting was significantly higher (*p <* 0.05). As IL increased, paternal depression and negative self-concept levels decreased significantly (*p <* 0.05). When SFL increased, maternal anxiety, depression, and somatization and paternal negative self-concept levels decreased significantly (*p <* 0.05). In regression analyses, maternal anxiety was significantly associated with irritability, depression with hyperactivity, negative self-concept with irritability; somatization with irritability and the presence of medical diease in mother and patient; hostility with hyperactivity. Paternal anxiety, depression, somatization, and hostility were associated with irritability; negative self-concept with irritability and social withdrawal. It is hoped these results contribute to a better understanding of the protective and risk factors of the psychopathology of parents of adults with ASD, a topic relatively poorly studied.

## Introduction

Autism Spectrum Disorder (ASD) is a neurodevelopmental disorder with symptoms that begin in early childhood and have lifelong effects, and so also significantly impacts the well-being of other family members^1^. Studies have shown that parents of individuals with ASD experience higher levels of stress compared to both parents of typically developing children and parents of individuals with non-autism developmental disabilities. The parents of children with ASD also have higher levels of depression, anxiety, hopelessness, burnout, and a greater susceptibility to mental health problems (MHP)^1–4^. Studies evaluating the MHP of parents of individuals with ASD have shown that various factors are associated with parental psychiatric symptoms (PS) level. These factors include the age, gender, intelligence level, behavioral problems, severity of autism symptoms, presence of co-occurring mental and physical illnesses, and independence level of the individual with ASD. Furthermore, parent-related factors, such as age, coping style, and social support have also been shown to have a significant impact on the psychological outcomes experienced by parents^5–7^.

Considering the complex and chronic nature of ASD, parents encounter different stressors at various stages of their child’s life due to changes in risk factors affecting both the child and the parents^8–10^. Therefore, the challenges faced by parents are observed not only during the individual with ASD’s childhood and adolescence but also throughout adulthood. Studies have reported that parents of adults with ASD are at risk of developing MHP^9^. However, upon reviewing the literature, the majority of studies evaluating the psychopathology of parents of individuals with ASD include children and adolescents and only a few studies including adults^7,11–14^.

Although parents of individuals with ASD have been shown to be vulnerable to many psychiatric disorders, a meta-analysis reported that the majority of studies assessing psychopathological symptoms in parents of individuals with ASD focused on only depression and anxiety symptoms^3^. In addition, fathers were under-represented in studies evaluating PS in parents of individuals with ASD, making up only 16.9% of the sample. Thus, it appears that fathers are often overlooked in many studies^3^. This also means that there is insufficient data on the MHP of both mothers and fathers of adult individuals with ASD. In the present study, the aim was to determine the PS level in parents of adults with ASD, to compare the symptom levels between mothers and fathers, to investigate the relationship between parents’ PS and variables such as age, autism severity, problem behaviors, independence, social functioning, and some parental factors, including age, education level, working status and the presence of parental medical problems. There is a lack of published studies investigating the relationship between the level of independence and social functioning in adults with ASD and parental psychopathology. However, when individuals with ASD reach adulthood, the level of social functioning and independence in daily life activities is key for their participation in society. Deficiencies in these areas cause adults to be dependent on their parents. Therefore, we hypothesize that the deficiencies in independence and social functioning of adults with ASD will lead to increased PS in parents.

## Methods

The study was conducted at the Kocaeli University, Department of Child and Adolescent Psychiatry. Approval for the research was obtained from the Kocaeli University Faculty of Medicine Ethics Committee, under project number 2022/333. The study was performed in accordance with the Declaration of Helsinki and all participants gave written informed consent. The study included the cases that had previously attended clinic, diagnosed with ASD (excluding Rett Syndrome according to DSM-IV), had reached the age of 18 years, and had consented to participate along with their parents. Cases were excluded if the parent was illiterate, had severe mental disorders (requiring inpatient treatment such as psychosis, mood disorder during manic episode, moderate or severe mental retardation) or physical illnesses (such as hearing or speech impairment that would interfere with communication), or if the parent could not be reached.

After reviewing patient records, patients and parents who met the inclusion criteria were contacted by phone and informed about the study. A total of 87 parents agreed to participate in the study. These patients and parents constituted the Kocaeli University adult ASD sample (KAAS) and were invited to follow-up. All cases included in the sample who agreed to participate and provided informed consent, along with their parents, underwent detailed clinical interviews with a child psychiatrist between January and June 2023. Five cases that did not meet the criteria for ASD (based on the criteria established in the study by Helt et al.^15^ and five cases who had only one living parent were excluded from the study to avoid confounding factors. Thus, the study was conducted with 77 patients and their parents.

### Measures

#### Socio-demographic Data Form

The form used to gather information on the socio-demographic and clinical characteristics of the patient and their family was designed by the researcher based on existing literature. This form included questions about the patient’s age, gender, diagnosis, treatments received, family type, and age, education, and occupation of the parents, among other family-related characteristics.

#### Brief Symptom Inventory (BSI)

We administered the BSI to both parents to measure parental PS. The scale is a five-point Likert-type measuring tool which was developed by Derogatis to screen various PS^16^. The Turkish validity and reliability was conducted by Şahin and Durak^17^. This scale comprises five subscales and measures anxiety, depression, negative self-concept, somatization, and hostility. An increase in the total score from each subscale indicates an increase in the PS^18^.

#### Aberrant Behavior Checklist (ABC)

The ABC was developed to assess behavioral problems observed in individuals with neurodevelopmental disorders^19^. The scale consists of five subscales: irritability, social withdrawal, stereotypic behavior, hyperactivity, and inappropriate speech. An increase in the subscales scores indicates that the problem behavior severity is increased^19^. The Turkish validity and reliability was conducted by Sucuoğlu et al.^20^. The scale was designed to be filled out by any adult familiar with the patient. In the present study, the ABC was completed by the parents to assess behavioral problems. Due to the majority of cases included in our study either not having expressive speech or speaking in short phrases, the ‘inappropriate speech’ subscale was excluded.

#### Childhood Autism Rating Scale (CARS)

The CARS was developed to diagnose autism, assess its severity, and distinguish it from other developmental disorders^21^. The Turkish validity and reliability was conducted by Gassaloğlu et al.^22^ The total score of the scale ranges from 15 to 60. An increased score indicates an increase in symptom severity^21,22^. The scale was reported to be applicable across all age groups^21^ The CARS, initially designed as an assessment tool for children, has been shown through studies to be safely used in diagnosing, screening, and determining the severity of autism in adults^21,23^. Thus, in our study the CARS was used to evaluate the severity of autism in adult ASD cases.

#### Clinician Rated Severity of Autism Spectrum and Social Communication Disorders Scale

The scale was developed to measure the severity of ASD and Social Communication Disorder in adults according to the DSM-V criteria. The Turkish validity and reliability was conducted by Aydın et al.^24^. The scale is a two-item, four-point Likert-type assessment tool rated by the interviewer. The first item assesses the severity of social communication, while the second item evaluates the severity of restricted interests and repetitive behaviors, along with the support needs for these conditions^24^.

#### Clinical Global Impressions Scale (CGI)

The CGI was developed to assess the clinical course of all psychiatric disorders across all age groups^25^. The scale consists of three dimensions: illness severity, improvement and severity of side effects. In the present study, only the illness severity dimension was ued. Illness severity is evaluated on a scale from 1 to 7^25^.

#### Lawton Instrumental Activities of Daily Living (IADL)

The scale was developed by Lawton and Brody (1969)^26^. The Turkish validity and reliability study was conducted by Işık et al.^27^. The scale assesses eight complex activities of daily living; ability to use the telephone, shopping, food preparation, laundry, housekeeping, use of transportation, taking responsibility for own medication management, and ability to handle finances. If the person performs the activity independently, 3 points are awarded; if the person performs the activity with assistance, 2 points are awarded; and if the person cannot perform the activity at all, 1 point is awarded. An increase in the total score indicates an improvement in the individual’s ability to live independently^26^.

#### Social Functioning Scale (SFS)

The scale was developed to determine social functioning level of individuals in various domains^28^. The Turkish validity and reliability was conducted by Erakay (2001)^29^. The SFS consists of seven subscales: withdrawal, interpersonal behavior, pro-social activities, recreation, independence-competence, independence-performance, and employment/occupation. Each question on the scale is evaluated between 0 and 3 points. A higher score from each subscale indicates better functionality. The scale has two separate formats: one for the patient themselves and another for a caregiver to fill out^28^. In the present study, only the form intended for caregivers was used.

### Data analysis

Statistical analysis was conducted using the Statistical Package for Social Sciences, version 26.0 (IBM Inc., Armonk, NY, USA). Normality of the distribution was assessed using skewness and kurtosis values. In the present study, since the skewness and kurtosis values of the data were within ± 2, it was assumed that these variables met the normality assumption^30^. For descriptive variables, frequency and percentage distributions were used, while for continuous variables, mean and standard deviation values were employed. When comparing categorical data, the Chi-square test was utilized. Differences between groups were evaluated using the independent samples t-test. The relationship between scale scores was determined by using Pearson’s correlation analysis. Multiple linear regression analysis was used to assess the impact of variables related to the patient, mother, and father on BSI scores. In the regression analysis, all categorical variables were recoded as two categories (0 and 1) and a stepwise method was used to avoid the multi-colinearity problem due to the small sample size. For all analyses *p* < 0.05 was considered statistically significant.

## Results

**Fig. 1:**
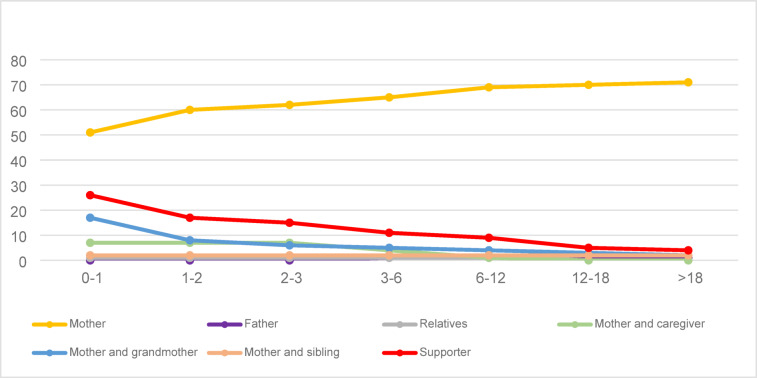
Change in proportion of care by caregiver category and individual with ASD age grouping.

The study included 77 individuals with ASD, aged between 18 and 39 years, with a mean age of 21.52 ± 3.16 years, along with their parents. When caregivers of the cases were queried, it was found that mothers were the primary caregivers most frequently across all age groups. During infancy and early childhood, caregivers included mothers, other relatives, and family elders. As individuals grew older, community support decreased, and mothers took on more caregiving responsibilities (Fig. 1).

The socio-demographic characteristics of the parents are summarized in Table 1. There was no difference in the education level between mothers and fathers. However, mothers had a significantly lower labor force participation rate compared to fathers (*p* < 0.001).

**Table 1:** Sociodemographic characteristics of the parents.

Variables		Mother (n=77)	Father (n=77)	*p*
Age (mean ±SD)		48.91 ± 5.23	52.83 ± 5.94	
		n (%)	n (%)	
Education level	Primary school or lower	32 (41.6)	20 (26.0)	0.155
	Secondary school	9 (11.7)	8 (10.4)	
	High school	21 (27.3)	32 (41.6)	
	University or higher	15 (19.5)	17 (22.0)	
Occupation	Housewife	60 (77.9)	–	<0.001*
	Retired	7 (9.1)	27 (35.0)	
	Civil servant	6 (7.8)	10 (13.0)	
	Worker	2 (2.6)	16 (20.8)	
	Self employment	2 (2.6)	24 (31.2)	
Medical disease	Yes	42 (54.5)	37 (48.1)	0.420
	No	35 (45.5)	40 (51.9)	

Chi-squared test, SD: standard deviation.

*p < 0.05.

Significant values are in [bold].

The ranking of PS levels derived from parents’ BSI scores were, from high to low: for mothers - depression, hostility, somatization, negative self-concept, and anxiety; for fathers - hostility, depression, negative self-concept, anxiety, and somatization. When comparing the PS of parents, fathers had higher hostility scores than mothers, while all other symptoms were higher in mothers. However, significant differences were observed in somatization and depression scores (Table 2).

**Table 2:** Parents’ Brief Symptom Inventory scores.

Variables	Mother (n=77)	Father (n=77)	t	*p*
	Mean ± SD	Mean ± SD		
Anxiety	0.50 ± 0.47	0.42 ± 0.45	0.973	0.332
Depression	0.91 ± 0.79	0.65 ± 0.60	2.218	0.028*
Negative self-concept	0.51 ± 0.58	0.50 ± 0.52	-0.127	0.899
Somatization	0.67 ± 0.62	0.38 ± 0.45	3.111	0.002*
Hostility	0.70 ± 0.57	0.73 ± 0.62	-0.260	0.795

Independent samples t-test, SD: standard deviation.

*p < 0.05.

Significant values are in [bold].

It was found that 45.5% of the cases with ASD had comorbid medical disease and 77.9% had comorbid psychiatric disorder. Furthermore, 77.4% of the cases used psychotropic medication and of these, 75.4% used multiple drugs. The somatization score in mothers of the cases with a comorbid medical diagnosis was significantly higher compared to mothers of the cases without a comorbid medical diagnosis (*p* = 0.033). The somatization score of the mothers of the cases with IQ below 70 was higher than the mothers of the cases with IQ above 70 (*p* = 0.011). Fathers of cases with IQ below 70 had significantly higher depression and negative self-concept scores compared to fathers of cases with IQ above 70 (*p* = 0.033 and *p* = 0.001, respectively). Fathers of cases who were dependent for self-care and toileting had significantly higher negative self-concept scores compared to fathers of cases who were independent in self-care and toileting skills (*p* = 0.008 and *p* = 0.018, respectively). Fathers of illiterate cases had significantly higher depression and negative self-concept scores compared to fathers of literate cases (*p* = 0.046 and *p* = 0.029, respectively) (Table 3).

**T﻿able 3:** Sociodemographic, clinical and functional characteristics of the cases and comparison of these variables with parental psychiatric symptom severity.

				Brief symptom ınventory									
				Mother					Father				
Variables	Group	n (%)		Anxiety	Depression	Negative self-concept	Somatization	Hostility	Anxiety	Depression	Negative self-concept	Somatization	Hostility
Gender	Female	10 (13.0)	Mean (SD)	0.53 (0.37)	1.07 (0.65)	0.54 (0.39)	0.79 (0.71)	0.83 (0.62)	0.39 (0.41)	0.70 (0.61)	0.50 (0.54)	0.57 (0.66)	0.65 (0.67)
	Male	67 (87.0)		0.54 (0.54)	0.97 (0.85)	0.55 (0.67)	0.71 (0.66)	0.72 (0.60)	0.48 (0.53)	0.69 (0.67)	0.56 (0.61)	0.43 (0.60)	0.82 (0.74)
			t (*p*)	0.035 (0.972)	-0.368 (0.714)	0.038 (0.97)	-0.358 (0.721)	-0.559 (0.577)	0.520 (0.604)	-0.084 (0.934)	0.317 (0.752)	-0.665 (0.508)	0.723 (0.472)
Comorbid medical diagnosis	No	42 (54.5)	Mean (SD)	0.53 (0.52)	0.84 (0.72)	0.48 (0.57)	0.59 (0.58)	0.69 (0.54)	0.41 (0.42)	0.62 (0.61)	0.50 (0.57)	0.40 (0.56)	0.73 (0.72)
	Yes	35 (45.5)		0.55 (0.44)	1.18 (0.92)	0.63 (0.59)	0.90 (0.73)	0.80 (0.67)	0.54 (0.53)	0.79 (0.63)	0.62 (0.52)	0.52 (0.57)	0.88 (0.69)
			t (*p*)	-0.191 (0.849)	-1.898 (0.077)	-1.004 (0.319)	-2.175 (0.033*)	-0.769 (0.444)	-1.079 (0.284)	-1.115 (0.269)	-0.804 (0.403)	-0.835 (0.406)	-0.840 (0.403)
Comorbid psychiatric diagnosis	No	17 (22.1)	Mean (SD)	0.44 (0.44)	0.85 (0.73)	0.48 (0.60)	0.65 (0.60)	0.79 (0.63)	0.39 (0.57)	0.50 (0.52)	0.39 (0.44)	0.28 (0.36)	0.62 (0.72)
	Yes	60 (77.9)		0.57 (0.55)	1.03 (0.85)	0.57 (0.65)	0.75 (0.69)	0.72 (0.059)	0.49 (0.50)	0.76 (0.69)	0.61 (0.64)	0.52 (0.66)	0.86 (0.73)
			t (*p*)	-0.971 (0.335)	-0.894 (0.374)	-0.548 (0.585)	-0.612 (0.542)	0.505 (0.615)	-0.752 (0.454)	-1.523 (0.132)	-1.465 (0.147)	-1.535 (0.129)	-1.262 (0.211)
IQ level	<70	55 (71.4)	Mean (SD)	0.60 (0.56)	1.09 (0.89)	0.62 (0.71)	0.84 (0.73)	0.78 (0.65)	0.55 (0.57)	0.80 (0.74)	0.70 (0.66)	0.54 (0.70)	0.86 (0.81)
	≥70	22 (28.6)		0.41 (0.42)	0.77 (0.63)	0.40 (0.44)	0.50 (0.45)	0.65 (0.47)	0.33 (0.37)	0.50 (0.42)	0.30 (0.34)	0.30 (0.36)	0.69 (0.57)
			t (*p*)	1.603 (0.113)	1.699 (0.093)	1.543 (0.127)	2.620 (0.011*)	0.970 (0.335)	1.992 (0.05)	2.174 (0.033*)	3.432 (0.001*)	1.900 (0.061)	0.974 (0.333)
Functional speech ability	No	43 (55.8)	Mean (SD)	0.61 (0.61)	1.06 (0.84)	0.60 (0.69)	0.75 (0.65)	0.75 (0.58)	0.53 (0.59)	0.80 (0.76)	0.68 (0.71)	0.48 (0.70)	0.78 (0.82)
	Yes	34 (44.2)		0.46 (0.41)	0.90 (0.81)	0.49 (0.57)	0.69 (0.68)	0.73 (0.62)	0.40 (0.43)	0.58 (0.51)	0.43 (0.42)	0.42 (0.50)	0.82 (0.64)
			t (*p*)	1.341 (0.184)	0.910 (0.366)	0.766 (0.446)	0.387 (0.700)	0.175 (0.861)	1.057 (0.294)	1.488 (0.141)	1.834 (0.072)	0.425 (0.672)	-0.248 (0.805)
Independent self-care skill	No	44 (59.1)	Mean (SD)	0.60 (0.62)	1.08 (0.89)	0.61 (0.74)	0.77 (0.67)	0.80 (0.63)	0.56 (0.59)	0.83 (0.76)	0.73 (0.70)	0.47 (0.69)	0.86 (0.90)
	Yes	33 (42.9)		0.47 (0.39)	0.88 (0.74)	0.47 (0.49)	0.66 (0.66)	0.67 (0.56)	0.37 (0.41)	0.55 (0.51)	0.37 (0.40)	0.44 (0.52)	0.74 (0.52)
			t (*p*)	-1.110 (0.261)	-1.074 (0.286)	-1.044 (0.300)	-0.739 (0.462)	-0.960 (0.34)	-1.593 (0.116)	-1.893 (0.062)	-2.731 (0.008*)	-0.212 (0.833)	-0.700 (0.486)
Independent toileting skill	No	31 (40.3)	Mean (SD)	0.69 (0.67)	1.18 (0.90)	0.72 (0.77)	0.83 (0.66)	0.84 (0.64)	0.59 (0.64)	0.89 (0.85)	0.83 (0.79)	0.58 (0.81)	0.93 (0.96)
	Yes	46 (59.7)		0.45 (0.40)	0.87 (0.76)	0.44 (0.52)	0.66 (0.66)	0.68 (0.57)	0.40 (0.44)	0.59 (0.51)	0.41 (0.41)	0.39 (0.47)	0.73 (0.59)
			t (*p*)	1.776 (0.083)	1.672 (0.098)	1.926 (0.058)	1.129 (0.262)	1.166 (0.247)	1.322 (0.195)	1.66 (0.105)	2.488 (0.018*)	1.114 (0.273)	0.970 (0.339)
Literacy learning status	No	40 (51.9)	Mean (SD)	0.66 (0.64)	1.11 (0.89)	0.66 (0.71)	0.79 (0.69)	0.79 (0.61)	0.58 (0.62)	0.88 (0.79)	0.74 (0.73)	0.53 (0.71)	0.81 (0.84)
	Yes	37 (48.1)		0.44 (0.39)	0.88 (0.76)	0.45 (0.51)	0.67 (0.65)	0.69 (0.59)	0.38 (0.41)	0.55 (0.49)	0.41 (0.40)	0.39 (0.45)	0.80 (0.61)
			t (*p*)	1.818 (0.075)	1.231 (0.222)	1.493 (0.139)	0.797 (0.428)	0.758 (0.451)	1.535 (0.131)	2.052 (0.046*)	2.251 (0.029*)	1.009 (0.316)	-0.072 (0.942)

Independent samples t-test, SD: standard deviation.

*p < 0.05.

Significant values are in [bold].

The mean CARS score of the participants was 39.74 ± 11.02; the mean CGI score was 4.92 ± 1.86. As a result of the evaluation of the Clinician Rated Severity of Autism Spectrum and Social Communication Disorders Scale, which measures the severity of ASD according to DSM-V criteria, the mean social communication (SC) severity was 2.06 ± 0.93, and the mean restricted interests and repetitive behaviors (RIRB) severity was 1.79 ± 1.01. The relationships between mothers’ and fathers’ BSI scores with the scores of the scales used in the study is summarized in Table 4. There was no significant relationship found between maternal and paternal BSI scores and the patient, mother or father age.

A positive significant relationship was found between the maternal anxiety score and the irritability, social withdrawal, hyperactivity, and stereotypic behavior scores of ABC, CARS, CGI, SC and RIRB scores. A negative significant relationship was identified between the maternal anxiety score and the SFS total score, pro-social activities and recreation subscale scores. Paternal anxiety scores had a significant positive correlation with irritability, social withdrawal, hyperactivity, stereotypic behavior, CARS and CGI scores.

Maternal depression score was correlated positively and significantly with irritability, social withdrawal, hyperactivity and stereotypic behavior, CARS, CGI and SC scores. Furthermore, matenal depression score was negatively correlated with recreation score. Paternal depression score was correlated positively and significantly with irritability, social withdrawal, hyperactivity and stereotypic behavior, CARS, CGI, SC and RIRB scores and negatively and significantly with the IADL total score.

Maternal negative self-concept score was correlated positively and significantly with irritability, social withdrawal, hyperactivity and stereotypic behavior, the CARS, the CGI and SC scores. In addition to these parameters, paternal negative self-concept score was also correlated positively with RIRB scores. Paternal negative self-concept score was correlated negatively with the IADL and the independence-competence and interpersonal behavior scores of the SFS.

Maternal somatization score was correlated positively with irritability, social withdrawal, hyperactivity and stereotypic behavior, CARS, SC and RIRB scores and negatively with recreation score. Paternal somatization score was correlated positively only with irritability score.

Maternal hostility score was correlated positively with irritability, social withdrawal, hyperactivity and stereotypic behavior scores. Paternal hostility score correlated positively with irritability score.

**Table 4:** Correlation analysis results between parents’ BSI scores with scale scores.

Variables		Brief Symptom Inventory									
		Anxiety		Depression		Negative self-concept		Somatization		Hostility	
		M	F	M	F	M	F	M	F	M	F
ABC	Irritability	.575**	.461**	.532**	.411**	.488**	.512**	.451**	.390**	.365**	.288*
	Social withdrawal	.402**	.303**	.469**	.234*	.378**	.257*	.413**	.162	.340**	.163
	Hyperactivity	.542**	.319**	.557**	.321**	.474**	.381**	.435**	.197	.396**	.173
	Stereotypic behavior	.467**	.384**	.439**	.284*	.412**	.324**	.426**	.219	.340**	.199
CARS		.265^*^	**.232** ^*^	**.256** ^*^	**.309** ^**^	**.231** ^*^	**.336** ^**^	**.229** ^*^	.150	.187	.095
CRSS	Social communication	**.300** ^**^	.199	**.295** ^**^	**.311** ^**^	**.234** ^*^	**.320** ^**^	**.254** ^*^	.169	.188	.083
	Restricted interests and repetitive behaviors	**.233** ^*^	.228	.186	**.258** ^*^	.158	**.308** ^**^	**.233** ^*^	.185	.176	.061
CGI		.281*	.243*	.247*	.302**	.224*	.340**	.208	.167	.160	.089
IADL total score		− .200	− .162	− .144	− .235*	− − .136	− .278*	− .137	− .085	− .101	− .032
SFS	Total score	− .242*	− .157	− .194	− .196	− .201	− .224	− .176	− .057	− .152	.033
	Withdrawal	− .121	− .119	− .094	− .068	− .075	− .150	− .079	− .108	− .027	− .072
	Pro-social activities	− .243*	− .170	− .199	− .182	− .196	− .201	− .208	− .091	− .166	.023
	Recreation	− .260*	− .111	− .245*	− .132	− .213	− .140	− .236*	.012	− .208	.061
	Independence-competence	− .209	− .147	− .180	− .196	− .191	− .230*	− .162	− .037	− .133	.025
	Independence-performance	− .196	− .115	− .121	− .149	− .165	− .179	− .101	− .011	− .108	.039
	Employment/occupation	− .070	− .131	− .037	− .203	− .052	− .193	− .034	− .133	− .018	.063
	Interpersonal behavior	− .181	− .111	− .173	− .211	− .148	− .231*	− .083	− .069	− .094	− .020

Pearson Correlation Analysis, *p < 0.05, **p < 0.01, M: Mother, F:Father, ABC: Aberrant Behavior Checklist, CARS: Childhood Autism Rating Scale, CGI: Clinical Global Impressions Scale, CRSS: Clinician Rated Severity of Autism Spectrum and Social Communication Disorders Scale, IADL: Lawton Instrumental Activities of Daily Living, SFS: Social Functioning Scale.

Significant values are in [bold].

Multiple linear regression analyses was used to assess predictors of the parental PS levels. The stepwise method was used to avoid the multi-collinearity problem, which were not evident in the present study. Variables related to the case (age, gender, independent toileting skills, independent self-care skills, functional speech ability, literacy learning status, intellectual disability, medical and psychiatric comorbidity, psychopharmacological treatment status, and number of medications used), parents (age, education level (primary or secondary school / high school or higher), working status and medical diagnosis), and the variables showing significant correlations with each PS based on correlation analysis (Table 4) were included as independent variables in the regression models.

Maternal anxiety level was predicted by irritability (R²=0.331), depression level was predicted by hyperactivity (R²=0.311), negative self-concept level was predicted by irritability (R²=0.238); somatization level was predicted by irritability and the presence of medical diease in both mother and patient (R²=0.301) while hostility level was predicted by hyperactivity (R²=0.157) (Table 5).

**Table 5:** Regression analysis results for variables predicting maternal psychiatric symptoms level.

Dependent variable	Model	Independent variables	β	t	*p*	Results of Model Analysis		
						F	*p*	R^2^
Anxiety	Model 1	Irritability	0.026	6.287	<0.001	39.530	<0.001	0.331
Depression	Model 1	Hyperactivity	0.038	6.006	<0.001	36.074	<0.001	0.311
Negative self-concept	Model 1	Irritability	0.027	5.005	<0.001	25.053	<0.001	0.238
Somatization	Model 1	Irritability	0.026	4.514	<0.001	20.379	<0.001	0.203
	Model 2	Irritability	0.026	4.554	<0.001	13.713	<0.001	0.258
		Medical disease in mother (yes)	0.311	2.412	0.018			
	Model 3	Irritability	0.024	4.374	<0.001	11.178	<0.001	0.301
		Medical disease in mother (yes)	0.335	2.649	0.010			
		Medical disease in patient (yes)	0.279	2.189	0.032			
Hostility	Model 1	Hyperactivity	0.019	3.861	<0.001	14.908	<0.001	0.157

Paternal anxiety (R²=0.212), depression (R²=0.169), somatization (R²=0.152), and hostility (R²=0.083) levels was predicted by irritability. Paternal negative self-concept level was predicted by irritability and social withdrawal (R²=0.307) (Table 6).

**Table 6:** Regression analysis results for variables predicting paternal psychiatric symptoms level.

Dependent variable	Model	Independent variables	β	t	p	Results of model analysis		
						F	p	R^2^
Anxiety	Model 1	Irritability	0.021	4.403	<0.001	19.383	<0.001	0.212
Depression	Model 1	Irritability	0.024	3.826	<0.001	14.635	<0.001	0.169
Negative self-concept	Model 1	Irritability	0.027	5.062	<0.001	25.623	<0.001	0.262
	Model 2	Irritability	0.040	4.963	<0.001	15.694	<0.001	0.307
		Social withdrawal	0.019	2.125	0.037			
Somatization	Model 1	Irritability	0.021	3.591	0.001	12.896	0.001	0.152
Hostility	Model 1	Irritability	0.019	2.555	0.013	6.529	0.013	0.083

## Discussion

We believe the present study holds the distinction of being the first to comprehensively examine the PS of parents of adults with ASD in relation to various factors. In all age groups, from childhood to adulthood, the most common primary caregiver of patients diagnosed with ASD was their mothers; as children with ASD grew older, community support decreased and mothers took on more care responsibilities. Mothers’ labor force participation rate was significantly lower than fathers’. When PS levels were compared, mothers’ somatization and depression levels were significantly higher than fathers’. The somatization score of the mothers of cases with comorbid medical diagnosis and intellectual disability was significantly higher than those without. The depression score of fathers of cases with intellectual disability and illiteracy was higher. The negative self-concept scores of fathers of cases with intellectual disability, illiteracy, and who were dependent for self-care and toileting were higher. In regression analyses, maternal anxiety was significantly associated with irritability, depression with hyperactivity, negative self-concept with irritability; somatization with irritability, the presence of medical diease in mother and patient; hostility with hyperactivity. Paternal anxiety, depression, somatization and hostility were associated with irritability; negative self-concept with irritability and social withdrawal.

With the increase in the prevalence of ASD and life expectancy, it is anticipated that the number of adult with ASD will increase^31^. As the number of adults with ASD increases, the number of aging parents of adults with ASD will also increases. Aging parents of individuals with ASD face challenges with their children’s education, employment, social relationships, economic independence, and independent living as their children reach adulthood^9^. In addition, problems such as the stress and difficulty of caring for a child with ASD, the need to adapt and accommodate to the child’s needs, the difficulty of balancing the needs of the family and the marital relationship, and the difficulty of accessing services when individuals with ASD reach adulthood have been found to cause chronic and devastating effects on family mental health^1^. Although the effects of ASD on parents’ mental health during childhood have been studied frequently, there is limited information available regarding the challenges faced by parents of adult patients^32^. Moreover, the majority of studies evaluating the parents of adults with ASD have been conducted in Western countries^3^ Indeed, research has shown that cultural differences in the perception of autism may lead to varying reactions among parents, highlighting the importance of considering the cultural context in studies related to caregiver health^33^. In Turkey, there was one published study in this field, and this study only addressed the depression and burnout levels of parents^11^. Thus, it is hoped that the present study is a major contribution to the literature about PS of aging parents of individuals with ASD within the cultural context and resources of Turkey.

As the ASD patient progresses from childhood to older age, the roles of family and community will change over time. Family involvement is expected to dominate in the early years, school to be prominent in the school years, and community support to increase as adulthood is reached^34^. However, contrary to expectations, community support is considerably lower in adulthood; formal support and services available to autistic adults are considerably rarer and less accessible than to autistic children. This causes many families, whether living in low- or high-income countries, to continue to maintain their primary caregiver roles and create their own informal care systems within the nuclear and extended family^34,35^. The parents of adult with ASD experience a contraction in their social support systems and the caregiver burden remains constant or increases, as was the case in the present study. Families of individuals with ongoing autism symptoms require support from their surroundings, often leading them to live close to or together with relatives^36,37^. Similar to the literature, in the present study, an extended family system was established, especially in the early years and extended family members assisted the mother, who was the primary caregiver. However, as the child grew older, the caregiving support from the surroundings decreased and the care burden was increasingly shouldered by the mothers. It was also observed that mothers were the primary caregivers in all age groups from childhood to adulthood. Although it has often been reported that mothers were most likely to be primary caregivers in studies evaluating the caregivers of children with ASD, knowledge about caregivers in adulthood is limited^38,39^. The present study, which illustrates the course of the caregiver burden throughout life in a Turkish population, shows that the mother remained the keystone in care responsibility, and even took more responsibility, in adulthood. Chronic care of a child with ASD causes caregiver burden and stress. It has been reported that the presence of social support for the parents of children with ASD reduces parental stress and helps them cope with the caregiver burden^37^. Similiary, in a study evaluating the parents of adult ASD cases, it was reported that informal social support was associated with caregiver burden and the quality of life of the parents, and that social support networks should be developed to support aging parents of adult children with ASD^36^. The importance of respite care services in this respect should be considered. A review found that families were the largest respite care providers for parents of children with ASD, and that formal care services were inadequate and inaccessible^40^. In this context, to alleviate caregivers burden and enhance their well-being, there is a need for specialized facilities and organizations focusing on the care and rehabilitation of adults with ASD, which are currently very scarce. This includes day care centers where families can receive short-term care support according to their needs.

Due to the lifelong nature of autism, the responsibility of caregiving and meeting the needs of a child with ASD has a constant effect on parents’ careers and employment. During the diagnosis and treatment process of ASD, parents face various challenges, such as leaving their jobs, retiring early, starting to work due to financial constraints, and transition to part-time work^41^ The literature shows that having a child with autism affects parents’ employment decisions and that mothers take more responsibility than fathers in taking care of their children by giving up employment^42,43^. In a study comparing the family function in parent of children with ASD and parents of typically developing children, it was reported that the employment rate of mothers of children with ASD was significantly lower than that of mothers of typically married children, while there was no difference among fathers^39^. In our study, it is noteworthy that although the educational levels of parents were similar, mothers’ participation in the workforce was significantly lower than fathers’. As a result of interviews with parents and our findings, it was seen that caregiving for children with ASD was predominantly managed by mothers, with many mothers sacrificing their careers to care for the child and to manage their child’s treatment. The mental health of mothers, who often take on care responsibilities alone, is also negatively affected by their leaving work, staying at home and increasing their responsibilities. This finding underscores the necessity for parents, especially the mothers of children with ASD, to have supportive measures to participate in the workforce. Insufficient support for parents leads to difficulties in finding employment opportunities and subsequent financial problems, being confined to home environments with their child, and an increase in MHP. Considering the challenges mothers face and the positive impact of employment on their mental well-being, it is important to find mechanisms to integrate these mothers into the workforce. It would be beneficial for public institutions, in particular, to create new job opportunities for mothers of individuals with ASD and to offer different work options, such as remote or flexible working arrangements.

From a family systems perspective, it is known that having an individual affected by a condition like ASD impacts the entire family. Therefore, we evaluated the PS of mothers and fathers separately, considering that it is important to evaluate both parents to understand family functioning. In the present study, mothers had significantly higher depression and somatization scores than fathers. However, there were no significant differences between parents anxiety, negative self-concept, and hostility levels. In the literature, there are differences comparing the PS level of mothers and fathers^3,4^. When reviewing publications that assess the PS of both parents, Gau et al. (2012) reported significantly higher somatization scores for mothers compared to fathers, similar to our findings^39^. However, Ou et al. (2010) reported no significant difference in somatization levels between mothers and fathers^44^. When examining anxiety levels, similar to our findings, Davis and Carter (2008) reported no significant difference between mothers and fathers^45^. Durukan et al., Foody et al., Gau et al., Ou et al. and Hastings reported that mothers had higher anxiety level^39,44,46–48^. Looking at publications that assess hostility levels, Ou et al. (2010) reported similar result with our study^44^. On the other hand, Gau et al. (2012) reported that mothers had a higher hostility level^39^. Similar to our study, many studies found that mothers’ depression scores were significantly higher than fathers’^11,38,39,45–50^. There was only one study that reported fathers’ depression scores to be significantly higher than mothers’^51^. Ou et al. (2010), Hastings (2003), and Benson (2006) reported no significant difference in depression levels between mothers and fathers^44,48,52^. Unlike other studies, Benson (2006) stated that all participating fathers were primary caregivers^52^. This situation suggests that when fathers take on the role of primary caregiver, they may experience similar PS levels as mothers. Therefore, it is thought that the difference in the findings is affected by whether the parents are primary caregivers or not.

When looking at the limited literature regarding fathers of children with ASD, when compared to mothers fathers spend less time on the care of their children, play a more indirect role in caregiving, and have less interaction with the child^53^. In studies evaluating the parents of individuals with ASD, it has been reported that mothers experience higher parenting stress compared to fathers, and this is considered a risk factor for the development of maternal psychopathology^3^ However, in a study evaluating the functioning of families with children with ASD, it was found that when overall caregiving responsibilities are evenly distributed, both mothers’ and fathers’ parenting stress was lower^54^. In the present study, the primary caregivers were mostly mothers, and maternal depression and somatization scores were significantly higher than fathers. This supports the hypothesis that primary caregivers of individuals with ASD, regardless of gender, are at higher risk for psychiatric disorders. The results of the present study, along with many other published studies, indicates that mothers are predominantly the primary caregivers of individuals with ASD, and their higher psychological distress is attributed to their greater responsibility in caring for their children. Considering the positive contributions of high involvement from both parents in the follow-up and treatment process of children with ASD to the overall family system, it would be beneficial to families to increase paternal involvement in caregiving.

The PS levels of parents of individuals with ASD has been shown to be associated with various case-related and parent-related factors^5–7^. As children with ASD grow up, parents are confronted with different stressors as a result of changes in risk factors^8–10^. There are few studies evaluating the impact of these factors when individuals with ASD reach adulthood. Findings from studies assessing parents of children and adolescents with ASD report differences depending on patient age. In longitudinal studies evaluating mothers of individuals with ASD, it was reported that there was no significant change in depressive symptoms as cases grow older, but a decrease in anxiety was noted^7,55,56^. We did not observe significant differences in the PS level in mothers and fathers with increasing age of cases, nor did we find significant differences based on the gender of the cases. This result suggests that parenting a child with ASD may lead to MHP in parents, regardless of the age and gender of the patient.

When individuals with ASD reach adulthood, the level of social functioning and independence in daily life activities becomes very important. A majority of adults with ASD continue to require support in daily life activities, leading dependent lives even in adulthood and resulting in a dependent lifestyle, which places a caregiving burden on their parents^57,58^. However, there is no study that examines the relationship between the level of independence and social functioning of adults with ASD and the PS level in their parents. We observed that as the independence level increased, there was a significant decrease in paternal depression and negative self-concept levels. As the SFS total score and pro-social activities score increased, maternal anxiety level significantly decreased. Moreover, as recreation score increased, maternal anxiety, depression, and somatization level decreased. It has been determined that as the independence-competence score, which measures the ability to fulfill the necessary skills for independent living, and the interpersonal behavior score, which assesses the quantity and quality of relationships with friends and others increased, paternal negative self-concept level decreased. Our research indicates that as the independence and social functioning levels of adults with ASD increase, the parental PS level decreases. In Turkey, the number of centers where adults with ASD can receive support is very limited. Therefore, in our opinion there is an urgent need to provide skills training aimed at improving the social skills and independence levels in daily life activities of adults with ASD, and to establish centers where they can apply these skills.

In the correlation analysis of the present study, the parental PS levels were mainly related to the severity of autism symptoms and problem behaviors. The irritability level of the individual with ASD predicted the maternal anxiety, negative self-concept and somatization levels and the paternal anxiety, depression, negative self-concept, somatization and hostility levels; the hyperactivity level predicted the maternal depression and hostility levels; the social withdrawal level predicted the paternal negative self-concept level; the presence of a medical diagnosis of the mother and the patient predicted the maternal somatization level. These findings support previous research indicating that the severity of problem behavior predicts the parental PS levels^4,7,55^. Follow-up studies reported that despite high rates of persistence in core symptoms of autism and accompanying behavioral and emotional problems, improvements have been observed in adulthood^10,59^. Our findings support that while there are changes in the severity of autism and accompanying problem behaviors in adulthood, the severity of problem behaviors remains a key predictor of parental PS. As individuals with ASD transition into adulthood, social life becomes increasingly important. Considering that problem behaviors may be socially inappropriate, it is likely that they contribute to less social integration and so higher levels of psychological burden on parents. Furthermore, during the period leading up to adulthood, parents may come to accept autism itself, yet the outwardly expressed problem behaviors rather than the core symptoms of autism could be perceived as exacerbating parents’ PS.

In a meta-analysis, it was shown that there was a bidirectional relationship between emotional and behavioral problems in individuals with ASD and parental MHP^5^. Parental MHP can restrict their ability to respond sensitively and consistently to the emotional needs of individual with ASD, to set boundaries, and to demonstrate positive parenting skills. This situation can increase internalized behavioral problems in children, such as withdrawal and avoidance, and externalizing behaviors like aggression and impulsivity. Likewise, an increase in autism symptoms and behavioral problems also predicts deterioration in parental mental health^5,60^. Considering the impact of parental MHP on family dynamics and children, early identification of parental PS, early diagnosis of psychiatric disorders, and timely treatment are of paramount importance. This approach not only enhances the health of parents but also prevents potential adverse effects of these symptoms on the prognosis of ASD. Therefore, the evaluation of parental PS should be integrated into routine monitoring procedures during the follow-up and treatment of individuals with ASD.

Furthermore, given the reciprocal relationship between parental MHP and emotional and behavioral problems in individuals with ASD, the importance of family-based interventions becomes evident. It has been shown that adding parent training to treatment programs for individuals with ASD not only positively impacts the child and family’s life but also enhances treatment outcomes^2^. Thus, it is generally considered important to implement appropriate intervention programs for parents. These programs should include psychoeducation about ASD following the diagnosis, provision of psychological counseling services, and social support programs that involve the entire family.

Our study has some limitations. Firstly, our sample consists of cases who have previously attended our clinic, and so our study group does not represent the entire population. Our cross-sectional study predominantly comprised young adults, similar to studies in the literature. However, characteristics across different stages of adulthood have not been investigated. Therefore, to generalize our findings, there is a need for multi-center longitudinal studies with larger sample sizes. In addition, we did not include a control group. Moreover, variables related to both the cases and their parents were assessed using scales filled out by the parents themselves, which may introduce bias due to potential inaccuracies in reporting. For future studies, it would be beneficial to add clinical interviews along with self-report scales to assess the PS level and psychiatric disorders of parents. Evaluating the prevalence of psychiatric disorders rather than just the severity of symptoms could provide valuable insights. Given the increasing number of adults with ASD and the lack of publications evaluating their aging parents, comprehensive new studies are needed to understand parental challenges and to identify support strategies for families. The strengths of our study include being one of the few studies that evaluate the PS of parents of adults with ASD, including both parents in the study, and assessing the relationship between the social functioning and independence levels of the individiuals and the parental PS level.

## Conclusion

Our study, evaluating the parents of adults with ASD demonstrated that the severity of problem behavior in adults with ASD strongly predicts the PS level in their parents. In previous studies, a bidirectional relationship has been reported between parental MHP and emotional and behavioral problems of individuals with ASD. Given these findings, appropriate interventions aimed at improving problem behavior will have positive effects on both the prognosis of the adult with ASD and the mental health of their parents. Considering the positive impact of parental mental well-being on individuals with ASD, it would be beneficial during the follow-up process of these cases to simultaneously assess the mental status of aging parents. Providing psychological support to parents and to implement family-based interventions would facilitate the psychological improvement of both patient and parents. This study has contributed to a better understanding of both protective and risk factors in the etiology of psychopathology in parents of older individuals with ASD. It is hoped that these findings stimulate further studies in this field in different populations and cultures. When our findings are confirmed and validated in other populations they will be beneficial for the planning and implementation of psychological support services for parents of adults with ASD.

## Data Availability

The datasets used and/or analysed during the current study available from the corresponding author on reasonable request.

## Declarations

### Author's Statement

A related but independent study, using a broader sample from the same cohort and focusing on social functioning and activities of daily living in individuals with autism, has been published, offering complementary and distinct insights compared to previous work (doi:https://urldefense.com/v3/__https://doi.org/10.1007/s10803-025-06969-8__;!!NLFGqXoFfo8MMQ!qqknXB-2OV8CyDhcshfuW92PEztExM7bSY7KoWfK3J9ExNb8rhO306gasoZsHVJEN4KRwzHmxYeTMvI-74nKpkVUhk8-t7s5HOqngaOBf9T8$).
